# Health outcomes and health-seeking behaviour following traumatic brain injury among older people: a prospective cohort study in Bangladesh

**DOI:** 10.3389/fragi.2025.1513137

**Published:** 2025-10-13

**Authors:** Farah Naz Rahman, Sukriti Das, Mohammad Rocky Khan Chowdhury, Manzur Kader, Saidur Rahman Mashreky

**Affiliations:** ^**1**^ School of Public Health and Preventive Medicine, Monash University, Melbourne, VIC, Australia; ^2^ International Centre for Diarrhoeal Disease Research Bangladesh (icddr,b), Dhaka, Bangladesh; ^**3**^ Department of Neurosurgery, Bangabandhu Sheikh Mujib Medical University, Dhaka, Bangladesh; ^**4**^ Department of Medical Science, School of Health and Welfare, Dalarna University, Falun, Sweden; ^**5**^ Centre for Injury Prevention and Research Bangladesh (CIPRB), Dhaka, Bangladesh; ^**6**^ Department of Public Health, North South University, Dhaka, Bangladesh

**Keywords:** traumatic brain injury (TBI), Road Traffic Injuries (RTI), older People, Bangladesh, LMIC

## Abstract

**Background:**

Older adults are at high risk for traumatic brain injury (TBI), yet there is limited evidence on their vulnerability to mortality, morbidity, and associated risk factors in low-and-middle-income countries. This study assessed the burden, health outcomes, and health-seeking behavior of TBI in older adults at the largest teaching hospital in Bangladesh.

**Methods:**

The study analyzed data from individuals aged 60+ years who were part of a prospective observational cohort of TBI patients admitted to a teaching hospital in Dhaka, Bangladesh, from May to June 2017. Data were collected at admission and during discharge or a 30-day follow-up (whichever came earlier) using a pre-tested semi-structured questionnaire, including the Glasgow Coma Scale (GCS), Glasgow Outcome Scale (GOS), and EuroQol-5D-3L. Descriptive analyses assessed the burden, characteristics, and health-seeking behavior for TBI, while relative risks were calculated to evaluate the risk of mortality by socio-demographic characteristics and clinical status.

**Results:**

During the study period, 117 older TBI patients were admitted, with 78.6% being male. Road traffic injuries (RTI) accounted for 71.3% of cases, followed by falls (16%). Half of the patients did not receive treatment at the primary and secondary facilities they initially visited, and 16% experienced over 24 h’ delay in treatment initiation. On admission, 25% presented with severe injury (GCS ≤8), and all had a history of loss of consciousness. The mortality rate was 5.2 per 1,000 person-days. Severe mobility issues and anxiety/depression were reported by 11% during follow-up. Bivariate analysis indicated higher mortality risk in patients with low socio-economic status, GCS ≤8, and over 1-h duration of both loss of consciousness and post-traumatic amnesia.

**Conclusion:**

RTI and falls are major causes of TBI, disproportionately affecting older adults of lower socio-economic status. Treatment accessibility gaps exist, and clinical status at admission is critical for predicting mortality. Findings can inform policies for preventive and rehabilitative strategies, including priority management protocols for older TBI patients in Bangladesh.

## 1 Introduction

Traumatic Brain Injury (TBI) among all injury types is considered to be the most severe in terms of clinical management and often has life-altering consequences, which makes it a major public health problem globally (Puvanachandra and Hyder, 2009). The incidence rate of TBI has increased by about 10% over the last 3 decades (Guan et al., 2023). In 2019, an estimated 49 million people endured TBI of all causes worldwide (Guan et al., 2023). TBI also results in high rates of mortality, morbidity, and it often leads to lifelong permanent disability (Gardner et al., 2018). It accounts for seven million YLD’s (Year loss in disability) annually, estimated in 2019 (Guan et al., 2023). Furthermore, injuries to the brain create substantial economic difficulties and compromise the quality of life for the affected individual and family members and burden the healthcare system for a nation at large. The direct and indirect costs associated with TBI related deaths were estimated to be around 1.1 billion in the USA (Humphreys and Wood, 2013). In addition to creating high personal and social burdens, TBI consumes a high volume of healthcare resources both at the acute and rehabilitation stages (Humphreys and Wood, 2013). The treatment and recovery process for TBI can be very difficult and lengthy. Often a multi-system approach that targets both the physical and psychological damages incurred in such an incident is needed for a meaningful and sustainable recovery.

The burden of TBI is particularly predominant in low- and middle-income countries (LMIC’s) which deal with a large number of these cases and have inadequate and under-resourced healthcare infrastructures to provide acute care for these individuals and manage the long-term health consequences (Hyder et al., 2007). LMICs have a higher incidence rate of TBI compared to an estimated global incidence rate (Bryan-Hancock and Harrison, 2010) and a review article by Puvanachandra and Hyder reports that the Southeast Asian and Western Pacific countries face the highest overall burden of TBI (Puvanachandra and Hyder, 2009). Yet, there is a lack of evidence from these regions that identify the risk factors and consequences of TBI (Bryan-Hancock and Harrison, 2010). LMIC’s generally have a weaker social safety net and almost non-existent supportive systems to look after the TBI patients after they survive and try to integrate back to society. Further, the economies of the LMICs are primarily based on manufacturing, transportation, construction and agriculture which make the risk of sustaining TBI high at the same time make it very difficult for the TBI victims to reintegrate into the labour force. This calls for epidemiological and preventive research on TBI specific to LMICs as the evidence from the high-income countries may not be applicable there. Since TBI incidents are mostly preventable, data on epidemiological profile and pattern of TBI in LMICs are vital for the development and implementation of region and context specific prevention programs (Puvanachandra and Hyder, 2009).

The old age population has been identified previously as the second most vulnerable group for TBI, preceded by younger adults (Goodman and Englander, 1992). Studies reveal a bimodal trend where young adults and older adults have the highest rate of TBI in all levels of severities (Bruns and Hauser, 2003). With the fast growing worldwide aging population, the incidence of TBI is rising in this population group, which has increased by two-fold in the past 18 years according to one study (Mak et al., 2012). In many Western countries, the epidemiology of TBI is evolving with a growing proportion of critically ill older TBI patients admitted to Intensive Care Units (Stocchetti et al., 2012). The risk factors and consequences of TBI among older patients are likely different from younger patients. Age alone contributes to the increased risks of hospital admission following TBI (Peschman et al., 2011). For older patients, even mild TBI poses a significant mortality risk (Cheng et al., 2014). Further, older adults also have a greater need for long-term care and rehabilitation services in comparison to young and middle-aged patients (LeBlanc et al., 2006). Moreover, the financial burden for TBI treatment of older patients is also higher than in other age groups (Chan et al., 2009). For this group of the population, prompt admission and comprehensive management are even more important to have a better treatment outcome along with the quality of life (Papa et al., 2012).

Although older adults are a particularly vulnerable group, evidence on TBI among them remains scarce (Mak et al., 2012), especially in LMICs. The few research studies that have been conducted on TBI in the LMICs such as India, Sub-Saharan Africa, including Bangladesh, did not provide much information on older population-who are the most vulnerable (Agrawal et al., 2016; Tran et al., 2015; Hossain et al., 2018). A study in India’s Level-1 trauma centre reports higher mortality rates among >50 aged patients due to head injury, but no further substantial information has been provided on this group (Agrawal et al., 2016). Another study on a tertiary healthcare centre of Uganda which tried to explore the distribution and characteristics of TBI had very few old-age patients and therefore their vulnerability picture could not be depicted in the study (Tran et al., 2015). Similarly, In Bangladesh, there is a lack of age-specific TBI research. Since the lifestyle and health status are different in older adults than young people (Rahman et al., 2021a; Tasnim et al., 2024), risk factors and characteristics of the victims should be explored in-depth to develop and implement age-specific prevention programs and offer a better and may be a different clinical management plan for them. The current study was conducted to provide a comprehensive picture of TBI among older patients by investigating the causes, clinical characteristics, outcomes, and health-seeking behaviors of this population. Additionally, it investigated the association of clinical characteristics and sociodemographic profiles with health outcomes among this population.

## 2 Methods and materials

### 2.1 Study design, site, and population

A prospective observational cohort study was conducted at Dhaka Medical College and Hospital (DMCH), the largest government teaching hospital in Bangladesh, located in Dhaka (Rahman et al., 2025). All patients admitted to the emergency department of DMCH with a primary diagnosis of Traumatic Brain Injury from May 1 to 30 June 2017, were included in the study. The primary diagnosis was based on the initial attending doctor’s assessment using patient history and clinical findings. This study analyzed the data of patients aged 60 years and above.

### 2.2 Data collection and instruments

Data were collected through face-to-face interviews with patients or their attendants if the patient was unable to respond. Data were collected at two time points: upon admission and at discharge, or 30 days after admission, whichever occurred first. At admission, detailed socio-demographic information (age, sex, education, occupation, family structure, monthly income) and clinical characteristics (Glasgow Coma Scale [GCS], duration of loss of consciousness, duration of post-traumatic amnesia) were recorded. Additionally, information on the causes of TBI and initial health-seeking behaviors was collected. At discharge, outcome variables were assessed using the Glasgow Outcome Scale (GOS) and the EuroQol-5D-3L (EQ-5D-3L) questionnaire.

The GCS was used at admission to evaluate the patient’s initial clinical status. The GCS score is derived from eye, verbal, and motor response assessments, and categorized into three levels: 3-8 represents a severe head injury (coma), 9–12 indicates a moderate injury, and 13–15 reflects a mild injury. The GOS, utilized at follow-up, helped to determine the patient’s recovery outcome which ranged from death to good recovery. It assesses the functional outcome following a brain injury with five levels: one indicates death, two means a persistent vegetative state, three represents severe disability (conscious but dependent on others), four indicates moderate disability (independent but with some deficits), and five signifies good recovery (resumption of normal life with minor deficits). The GCS and GOS both are widely used instruments to clinically assess the condition of patients with TBI. Among the alive patients, EuroQol five domains three levels questionnaire (EQ-5D-3L) was also used during follow-up to gather information on health outcomes. EQ-5D-3L is a well-known, patient- or respondent-informed standardized indicator of health status (Instruments, 2020). For this study, the descriptive system of EQ-5D-3L was used which measures health condition in five domains (Mobility, self-care, usual activities, pain/discomfort, anxiety/depression), each of which has three stages of responses (no problem, some problem, extreme problem). The study involved registered medical practitioners to conduct the data collection, including administration of GCS, GOS, and EQ-5D-3L.

### 2.3 Case definition

Traumatic Brain Injury was defined in alignment with the International Classification of Diseases, 10th Revision (ICD-10). The diagnosis by the first attending clinician determined eligible cases. TBI was treated as a nature-of-injury, with data collected on the causes and outcomes of the injury. The patient’s clinical condition, including clinical death, was recorded based on both the attending clinician’s declaration in the medical record of the hospital, and evaluation by GOS and EQ-5D-3L during follow-up.

### 2.4 Data analysis

Descriptive analysis was conducted with STATA to explore socio-demographic characteristics, causes and pattern of injury, and health-seeking behaviour of older TBI patients. Relative risk was calculated to explore the associations between socio-demographic factors and clinical presentations with TBI outcomes, with outcome variables including the patient’s status (dead or alive) at follow-up. Additional analysis included the chi-square test to examine the vulnerability of older TBI patients by exploring their susceptibility to poor outcomes compared to other age groups.

## 3 Results

The original cohort consisted of 659 TBI patients recruited over a 3-month study period, of whom 117 (17.8%) were aged 60 years and above and were included in this analysis. The following sections present the data for TBI patients aged 60 years and above from this cohort.

### 3.1 Sociodemographic profile of the patients

The mean age of the patients was 67.8 years, and majority (78.6%) of them were male. More than half of them had no formal education (54.7%) and had a living arrangement with their children’s family (60.7%). Majority of them (73.5%) were not working at the time of data collection, where most males were retired and most of the females were housewives. About half (49.6%) of the respondents had family income within 10–20 thousand BDT (Bangladesh Taka) per month (equivalent to 118–235 USD). (Table 1).

**TABLE 1:** Sociodemographic profile of the older TBI patients at a tertiary care teaching hospital in Bangladesh.

Variables	Male N (%)	Female N (%)	Total N (%)
Age			
Up to 70 years	58 (80.6%)	14 (19.4%)	72 (61.5%)
70+ years	34 (75.6%)	11 (24.4%)	45 (38.5%)
Education			
No formal education	51 (79.7%)	13 (20.3%)	64 (54.7%)
Primary	18 (72%)	7 (28%)	25 (21.4%)
Secondary and above	23 (82.1%)	5 (17.9%)	28 (23.9%)
Occupation			
Currently not working/Retired/Housewives	72 (83.7%)	14 (16.3%)	86 (73.5%)
Farmer	11 (57.9%)	8 (42.1%)	19 (16.2%)
Business/Service/other skilled work	9 (75%)	3 (25%)	12 (10.3%)
Family Structure			
Single family	32 (84.2%)	6 (15.8%)	38 (32.5%)
Joint family	60 (84.5%)	11 (15.5%)	71 (60.7%)
Family Income per month (in BDT)			
10 thousand and less	37 (82.2%)	8 (17.8%)	45 (38.5%)
10 to 20 thousand	46 (79.3%)	12 (20.7%)	58 (49.6%)
More than 20 thousand	9 (56.3%)	7 (43.8%)	16 (13.7%)

### 3.2 Causes and characteristics of the traumatic brain injury

**FIGURE 1:**
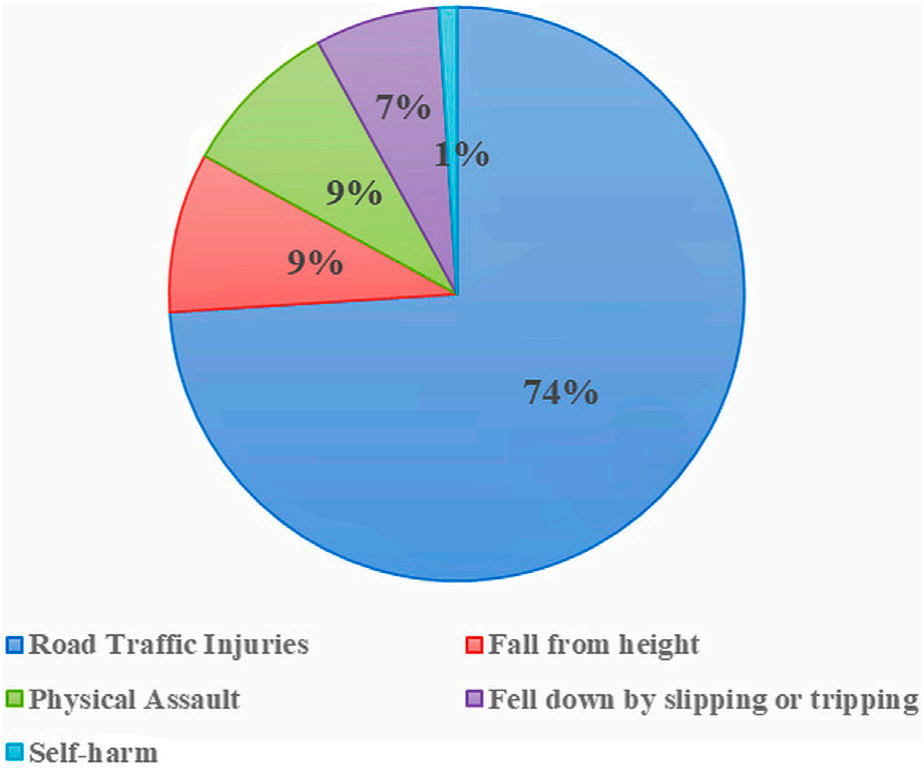
Events that led to TBI among older people in a tertiary care teaching hospital in Bangladesh.

Almost all (91%) of the injuries occurred accidentally/by unintentional means. Road Traffic Injuries (RTI) was found to be the leading cause of TBI among old age patients (74%, n = 87), followed by fall (16%, n = 19) from any level. Other causes of TBI in this group includes physical assault and self-harm (Figure 1).

The majority (63.2%, n = 55) of the patients who experienced RTI were pedestrians. One-fourth other were passengers (n = 23), and only few (10.5%, n = 9) were in the driving role. Nearly all (98.5%) of older TBI patients reported not using any safety measures during the incident. This included neglecting to use footpaths, foot over bridges, or safe crosswalks as pedestrians, as well as failing to wear seatbelts or helmets when they were passengers or drivers. Of the patients who got injured from falling (n = 19), more than half of them fell from height. Of the respondents who fell from height, most (60%, n = 7) got injured by falling from trees. Main road was predominant among the places where TBI among older patients occurred, followed by home environment. In home, incidents of TBI occurred mostly (63.6%) in bathroom.

### 3.3 Clinical presentation of TBI patients at admission

On admission, about one-third (31.6%, n = 37) of the TBI patients presented with severe injury with GCS score 8 or less, and half of the respondents presented with mild injury with GCS score 13 or more. All respondents reported experiencing loss of consciousness at some point after the injury, ranging from a few seconds to over 24 h; approximately 15% (n = 17) remained unconscious for more than 24 h. Further, a large proportion of patients (60.6%, n = 71) complained of suffering from post-traumatic amnesia (PTA) during admission. Although, in most of them, the PTA persists from few seconds to few minutes, 14.1% (n = 10) had PTA of more than 24 h’ duration. Also, about 30% (n = 34) patients had associated injuries with TBI, majority of which were limb injuries (Table 2).

**TABLE 2:** Clinical presentation of older TBI patients during admission at a tertiary-care teaching hospital in Bangladesh.

Variables	Number (n)	Percentage (%)
Glasgow Coma Scale (GCS)		
Mild (≥13)	42	35.9
Moderate (9–12)	38	32.5
Severe (≤8)	37	31.6
Loss of Consciousness		
Few minutes to 1-h	80	68.4
>1–24 h	20	17.1
More than 24 h	17	14.5
Post-Traumatic Amnesia (PTA) [N = 71]		
Few seconds to few minutes	49	69.0
1–24 h	12	16.9
More than 24 h	10	14.1
Associated Injury [N = 34]		
Spinal	3	8.8
Thoracic	6	17.6
Limb	17	50.0
Others	8	23.5

### 3.4 Health outcome of the older patients following TBI

Following admission, 15.4% (n = 18) of the older TBI patients needed surgical intervention and the rest were treated with conservative methods, i.e., medicine. Half of the surgical interventions involved debridement procedures and others included decompression, elevation of depressed fracture and burr hole.

**FIGURE 2:**
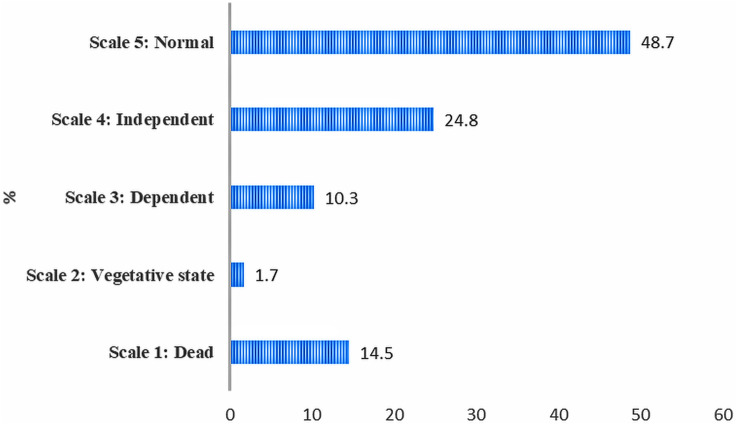
Distribution of glasgow outcome scale (GOS) outcomes among older TBI patients in a tertiary care teaching hospital in Bangladesh.

**FIGURE 3:**
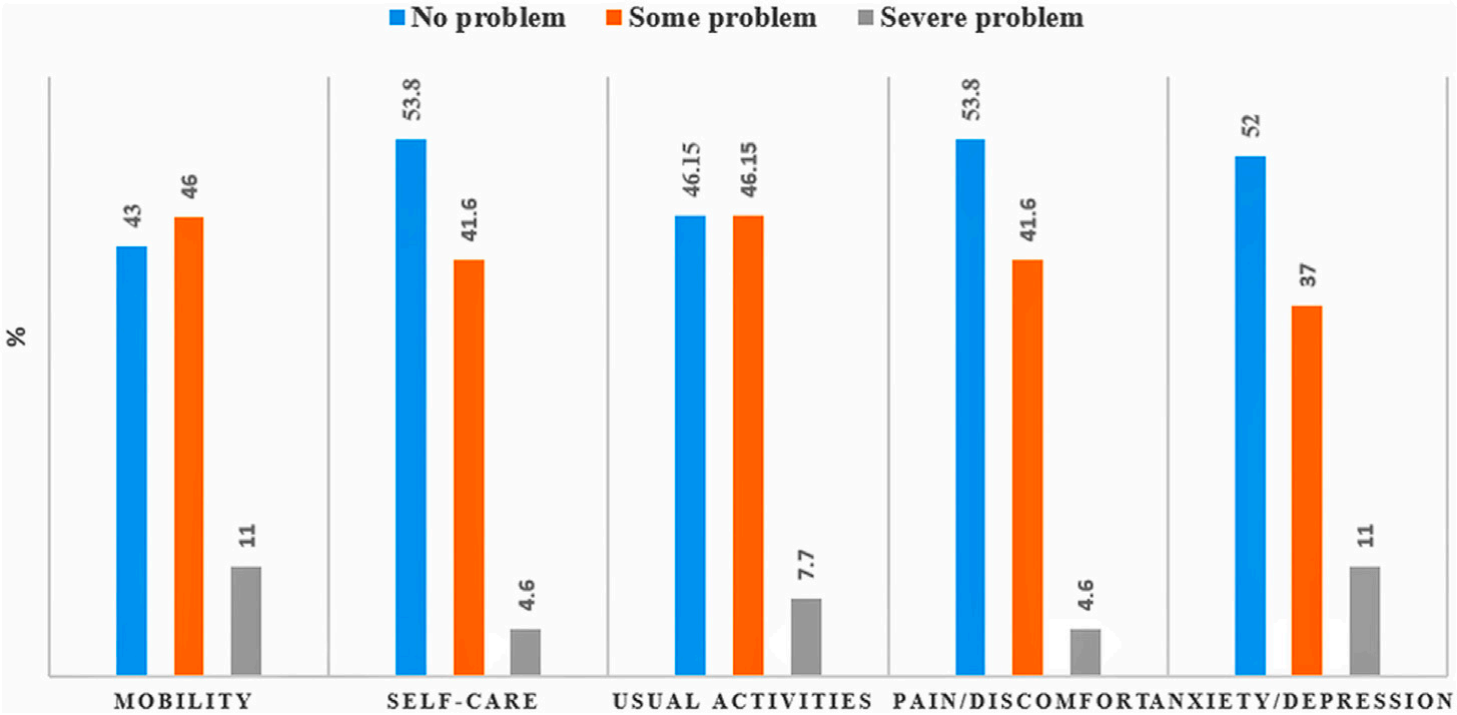
Reported levels of EQ-5D-dimensions of Older TBI Patients at a Tertiary-care Teaching-hospital in Bangladesh.

Mortality rate (within 30-days) for TBI among the older patients was 5.2 per 1,000 person-days (CI: 2.7–7.6) [n = 17]. According to GOS, almost half of the older patients had good recovery and one-fourth were able to carry on activities independently. (Figure 2). Furthermore, reported levels of EQ-5D-3L by older TBI patients (n = 100) during follow-up revealed that more than half respondents did not have any problem in maintaining self-care or did not suffer from any pain or anxiety. However, a considerable number (about 40%) reported to have some problem in all the five dimensions. Severe problem in mobility and severe anxiety/depression were reported by 11% of the respondents. (Figure 3). Moreover, at the time of discharge 21.5% of them registered some form of residual complications. PTA (35.7%) and communication and behavioural problem (42.8%) were predominant among the residual complications during discharge.

#### 3.4.1 Association between sociodemographic and clinical factors and mortality risk among older TBI patients

Table 3 presents the risk of mortality following TBI among older adults, categorized by sociodemographic and clinical factors. A significant association was observed between income level and mortality; individuals earning less than 10,000 BDT monthly had nearly three times higher mortality (24%) compared to those with higher incomes (8%), with a relative risk (RR) of 2.9 (95% CI: 1.2–7.3). Similarly, the severity of TBI, as indicated by the GCS score, showed a significant association, with patients experiencing severe TBI facing a mortality rate that was three times greater (27%) than those with mild to moderate injuries (9%), corresponding to an RR of 3.1 (95% CI: 1.3–7.5). Duration of loss of consciousness was also a significant factor, where those unconscious for over an hour had a 29% mortality rate, which was more than three times higher than the 9% observed in patients with shorter unconscious periods (RR: 3.3, 95% CI: 1.4–8.1). Additionally, extended post-traumatic amnesia was linked with increased mortality; individuals with amnesia lasting more than an hour had a rate of 32%, compared to 10% in those with shorter amnesia duration, with an RR of 3.1 (95% CI: 1.1–8.7). Conversely, no significant associations were found between mortality and factors such as gender or education level.

**TABLE 3:** Risk of mortality following TBI among older people in Bangladesh by sociodemographic and clinical status.

Variable	Outcome		RR	95% CI
	Alive, N (%)	Dead, (%)		
Gender				
Male	81 (88)	11 (12)	2	0.8–4.8
Female	19 (76)	6 (24)		
Education				
No formal education	55 (86)	9 (14)	0.9	0.3–2.2
Literate	45 (85)	8 (15)		
Income (monthly)				
Less than 10 thousand BDT	34 (76)	11 (24)	2.9*	1.2–7.3
10 thousand and more	66 (92)	6 (8)		
Glasgow Coma Scale				
Severe	27 (73)	10 (27)	3.1*	1.3–7.5
Mild to Moderate	73 (91)	7 (9)		
Duration of loss of consciousness				
More than 1 h	25 (71)	10 (29)	3.3*	1.4–8.1
Less than 1 h	75 (91)	7 (9)		
Duration of Post Traumatic Amnesia				
More than 1 h	15 (68)	7 (32)	3.1*	1.1–8.7
Less than 1 h	44 (90)	5 (10)		

*Statistically significant at p < 0.05.

### 3.5 Health-seeking behaviour of TBI patients

**FIGURE 4:**
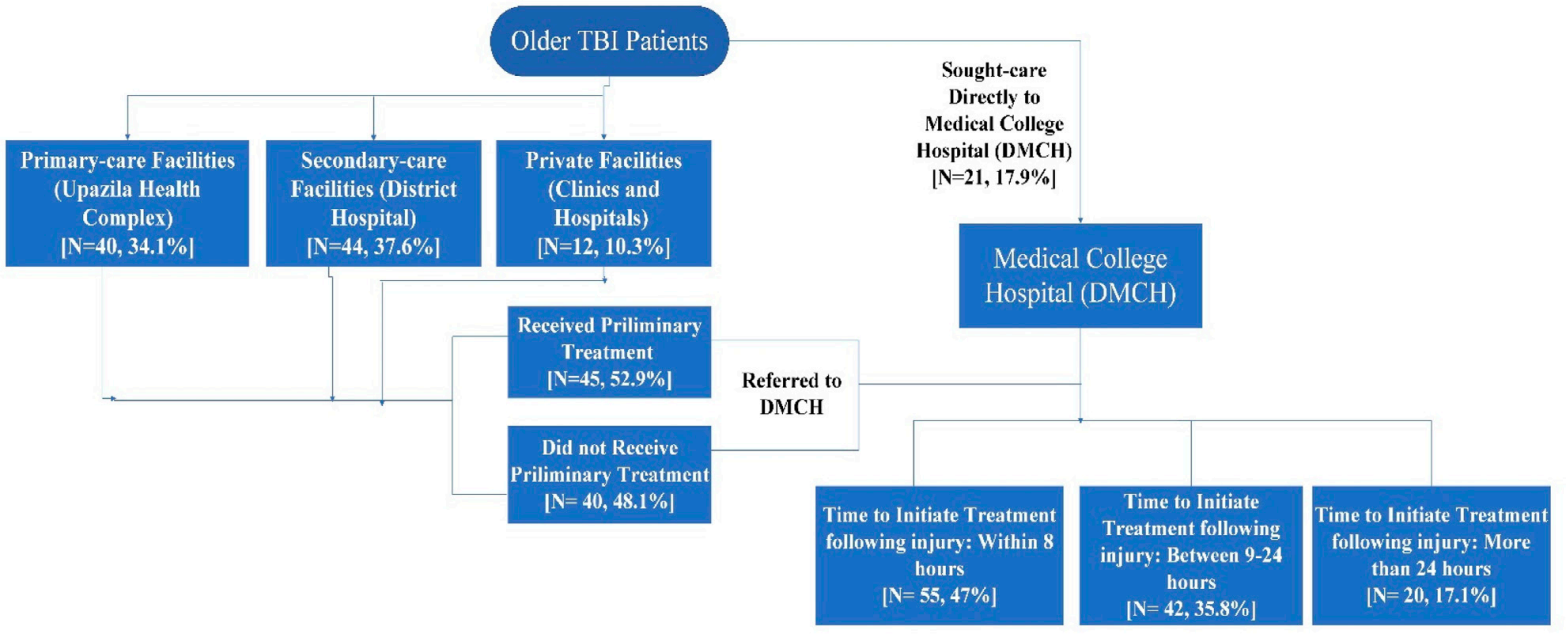
Health-seeking behaviour of older patients following traumatic brain injury (TBI) in Bangladesh.

Figure 4 presents the health-seeking behaviour of older patients after TBI. Following injuries, a substantial number (71.5%) of older patients sought care from local semi-urban primary to secondary level health facilities that are district hospitals (37.7%) and upazila health complexes (34.1%); from these facilities they were referred to Medical College Hospital (DMCH). Only 17.9% of the patients went directly to the DMCH for treatment. The remainder of the patients went to private facilities, and were referred to DMCH from there. Furthermore, of all patients who were referred from other hospitals, half of them reported receiving no treatment for their injury prior to referral. Majority (68.4%) of the older patients suggested their preference of first place of care was focused on the near distance from the location of the injury. Almost all (94.7%) older TBI patients reported that they did not receive any first aid treatment after the injury. Even though few received first aid services, none of their caregivers were trained in first aid emergency response. About 17% patients had more than 24 h’ delay in initiation of treatment from the time of injury. Further, more than one-third (35.8%) of the patients had a time gap between 9–24 h from occurrence of the injury to initiation of treatment. Those who got discharged within the 30-days follow-up, none of them received any rehabilitative management including psychosocial counselling.

## 4 Discussion

Although TBI research has expanded over the years, older adults remain underrepresented despite being a highly vulnerable group (Gardner et al., 2018). In Bangladesh, where injury rates are high, understanding TBI is particularly important as it is among the most severe forms of injury. This study is the first in Bangladesh to focus on older adults with TBI and one of the few from LMICs. It offers a comprehensive overview of the burden, key characteristics, associated risk factors, and health-seeking behaviors in this population.

Analyzing data from patients aged 60 and above within a larger cohort, the study found that approximately two out of every 10 TBI cases involved older adults. Likewise, studies in US found that older patients consist about 20% of the total TBI patients, and accounts for about 40% of the deaths due to TBI (Peschman et al., 2011; Waltzman et al., 2022). This implies a significant burden for older population group in Bangladesh which makes up 7.5% of its’ total population (Barikdar et al., 2016). Males are identified as predominant sufferer from TBI in general as culturally men are more exposed to outer world and responsible for wage earning in the family. The Bangladesh Health and Injury Survey (BHIS), 2016 also found that males, across all age groups, experienced head injuries more frequently than females. (Hossain et al., 2018).

The study also shows income to be significantly associated with the mortality outcome in older TBI patients. Similar findings are shared by several previous studies which tried to explore the impact of socio-economic status on the outcome of TBI patients (Haines et al., 2019; McQuistion et al., 2020). Even having treated with uniform treatment protocols in a public healthcare facility, older adults of low economic status experience poorer outcome; this may be explained by the presence of co-morbidities, poor nutritional status, inadequate hygiene and weak immunity in this group of people (Dhandapani et al., 2012). Beyond healthcare access and comorbidities, emerging research also suggests a biological underpinning linking low SES with worse neurocognitive and recovery outcomes after brain injury. Chronic psychosocial stress, often embedded in poverty, is associated with elevated levels of glucocorticoids and neuroinflammatory markers such as IL-6 and TNF-α, which are known to exacerbate neural injury and impede recovery after TBI (Muscatell et al., 2020). The concept of “psychosocial defeat”, common among individuals in structurally disadvantaged contexts, is implicated in chronic microglial activation and prolonged neuroinflammation, potentially worsening outcomes following neurotrauma (Kraynak et al., 2018).

Road traffic injuries were the predominant cause of TBI among older adults which is not a surprise for a country where road accidents claim on average about 12,000 lives annually (Alam, 2018; Rahman and Rahman, 2019). Another hospital-based survey in DMCH and a national survey on injury also found RTI to be responsible for majority of TBI among all ages in Bangladesh (Hossain et al., 2018; Mondol et al., 2013). The study also examined the role of older adults in RTIs, finding that most were pedestrians who lacked access to safe options such as footpaths, footbridges, or designated crosswalks. Similar findings have been reported in a North American rehabilitation clinic study, which identified pedestrian accidents as a common cause of TBI among older individuals (Goodman and Englander, 1992).

Falls were the second most common cause of TBI among older people in Bangladesh, accounting for about one-fourth of cases. In contrast, most studies from high-income countries identify falls as the leading cause of geriatric TBI (Goodman and Englander, 1992; Mak et al., 2012), highlighting a distinct pattern in LMICs. In an agricultural country like Bangladesh, falling from tree during fruit harvesting season is a big concern, and the study depicts similar picture as majority of the falls from height occurred by falling from the trees. Additionally, the study shows about 10% of the incidents occurred in home environment. This is important as older adults spend a considerable amount of time in home and it is generally considered to be a safe space for them. The incidents in home mostly occurred by falling or tripping on the ground and primarily took place in the bathroom. This suggests a lack of understanding and information about protective measures, which is also supported by the fact that none of the older adults used any safety measures such as gripping aid or anti-slip mat during those incidents. The mechanism of fall in older TBI patients in Bangladesh differ considerably from developed country scenario where most of the fall related injury results from sports or physical exercise related activities such as falls while skiing or bi-cycle riding (Gardner et al., 2018; Chehuen Neto et al., 2018). Although different in mechanism, the causes and nature of fall incidents in Bangladesh suggest that they are also largely preventable with proper awareness and prevention strategies (Rahman et al., 2021b). To prevent the fall incidents among older group, awareness regarding household risk factors such as low lighting, slippery floors, obstacles, long clothes, etc., and the practice of using safety tools such as anti-slip mat, safety bars, safety shoes and walking aid sticks can be beneficial (Chehuen Neto et al., 2018).

Older patients were found to have substantial adverse outcome both in terms of mortality and morbidity following TBI. CRASH (Corticosteroid Randomization after Significant Head Injury) data on global incidents also identified old age group as most vulnerable but evidence for Bangladesh was unavailable till date (Biswas et al., 2017). Several other studies also confer that outcome in old age group following TBI is worse than that of other age groups (LeBlanc et al., 2006; Papa et al., 2012). Advanced age is a recognized predictor of poor TBI outcomes, and our findings align with this evidence. Biological factors such as reduced neuroplasticity, diminished neurogenesis, and impaired cortical remapping limit recovery in older adults (Orr et al., 2015). Aging brains are also more susceptible to secondary injury mechanisms, including oxidative stress and mitochondrial dysfunction, which worsen prognosis even in mild TBI (Shively et al., 2012). These factors, combined with the lack of age-specific rehabilitation services in LMICs such as Bangladesh, underscore the urgent need for tailored geriatric TBI care and long-term management strategies.

About half of the older patients had moderate problem in all the five domains of EQ-5D-3L during discharge. Further, although more than half of the older respondents had moderate to severe difficulty in mobility at the time of discharge, no rehabilitative management or physiotherapy was given to them. Difficulty in movement and the need for intensive rehabilitative management for older TBI patients is also suggested by several other studies focusing outcome in geriatric patients (Goodman and Englander, 1992; LeBlanc et al., 2006; Alam, 2018). No psychosocial counselling was provided to any of the patients either despite about half of them had some to severe form of anxiety or depression. Similarly, a research report on geriatric TBI showed that depression is prevalent in around 50% of older TBI patients and often has a major effect on their quality of life (Papa et al., 2012). This also supports the fact that rehabilitation and psychosocial supports are generally unavailable for patients in most healthcare facilities of Bangladesh, even in a tertiary care centre, despite having a substantial demand (Mamin and Hayes, 2018; Islam, 2015).

This study also showed that health condition of older TBI patients at admission assessed by GCS ranking, length of lack of consciousness and PTA have independent influence on mortality. Further statistical analysis could not be performed due to the study’s small sample size. Comparable findings have been shared by other studies which also identified GCS score, length of coma and severity of injury as predictors of outcome in older TBI patients (Goodman and Englander, 1992; Stocchetti et al., 2012; Chan et al., 2009).

Moreover, older patients were primarily referred from their nearest secondary or primary healthcare facilities without receiving any prior treatment. The transportation and referral process eventually cause a long delay between injury and initiation of treatment. Waiting time in an overloaded public healthcare facility further delayed the initiation of treatment to more than 24 h in about one-fifth of older patients which have serious implications as prompt treatment in initial hours is vital after a head injury (Ashadullah et al., 2017). Nearly none of them received any first aid care either. All of these point towards an inadequate emergency care and a lack of trained first aid response team at root level or rural level where two-thirds of the country’s total population reside. Since there is a dearth of information regarding TBI among old age population in resource poor settings, these findings could not be compared with similar research.

### 4.1 Strengths and limitations

The study was conducted at the largest public teaching hospital in the country and usually only people from lower socioeconomic background attend this hospital. While this poses the limitation for single-institution based study, it is also one of the very few institutions which has functioning neurosurgical facility for patients in Bangladesh. It does draw and is attended by patients from all over the country, and the study results indicate that patients were referred from various primary or secondary health facilities across the whole country, thus enhancing the generalizability of the results. Another drawback of this study is that it has not reported the co-morbidities and behavioural risk factors that could have an effect on risk factors and outcome. There are, however, few research studies that indicated outcome of TBI on older group does not depend on comorbid conditions or behavioural factors (Stocchetti et al., 2012; Cheng et al., 2014). While mobility, anxiety or depression were assessed at discharge using EQ-5D-3L, the study did not capture baseline information on these conditions prior to injury. This limits our ability to distinguish pre-existing issues from injury-related outcomes. However, data collectors were trained to clarify whether these complaints emerged or persisted after the injury during EQ-5D-3L assessment, partly addressing this limitation. Moreover, although patients were followed up for 30 days, a longer follow-up period could have provided more comprehensive insights into the long-term disability, mortality, and other consequences associated with TBI, offering a deeper understanding of its outcomes. Additionally, since the data were analyzed from a larger cohort that did not exclusively focus on geriatric TBI, the study had to contend with a limited sample size, which constrained the scope for advanced and robust statistical analysis. Stratified analysis by injury severity to assess independent effects of factors like PTA and loss of consciousness could have provided deeper insights, but was not feasible due to the small sample size.

Despite the aforementioned limitations, this study is likely the first to explore geriatric TBI cases in Bangladesh using a prospective follow-up approach to examine outcomes and associated factors in this population. It provides a comprehensive view not only by highlighting the burden and associated factors but also by offering insights into the health-seeking behavior of older TBI patients treated at the country’s largest tertiary care center. This research establishes a valuable evidence base for further investigation and intervention studies on geriatric TBI in Bangladesh. Future research could benefit from larger sample sizes and extended follow-up periods to assess long-term disability and mortality. Additionally, exploring other outcomes, such as social and economic impacts alongside health outcomes, and considering the influence of comorbidities on these outcomes, could provide a more holistic understanding of geriatric TBI.

## 5 Conclusion

Road Traffic Injuries (RTIs) and falls are the leading causes of TBI in Bangladesh, disproportionately affecting older males from low socio-economic backgrounds. Including general road safety measures, specific safety programs such as educating the older group on safe pedestrian’s strategies and local collision hotspots, and make them aware on their functional limitations and physical vulnerability that needs to be considered while using road, is critical to prevent RTI in this group. Preventive measures in home regarding fall of older adults can be largely beneficial as well. The association of poor socio-economic condition with mortality among older TBI patients demands further in-depth research and actions such as increase awareness building in this group. In a resource-poor setting like Bangladesh where healthcare system is mostly overburdened, the value of predictive clinical status for outcome at admission needs to be further explored for proper triage and prompt case management. Aggressive treatment needs to be ensured in this older population group with separate geriatric ward, tailored treatment protocol, dedicated institution and management facility which is currently minimal to missing in Bangladesh. Mostly, primary and secondary healthcare facilities of rural Bangladesh need to be upgraded with appropriate emergency services to address TBI and a timely referral system should be in place. Comprehensive multi-system approach targeted to the old age group is essential to reduce this preventable burden of traumatic brain injury in Bangladesh.

## Data Availability

The original contributions presented in the study are included in the article/Supplementary Material, further inquiries can be directed to the corresponding author.
